# Prevalence of Vestibular Disorders in Independent People Over 50 That Experience Dizziness

**DOI:** 10.3389/fneur.2021.658053

**Published:** 2021-05-20

**Authors:** William V. C. Figtree, Jasmine C. Menant, Allan T. Chau, Patrick P. Hübner, Stephen R. Lord, Americo A. Migliaccio

**Affiliations:** ^1^Balance and Vision Laboratory, Neuroscience Research Australia, Sydney, NSW, Australia; ^2^Falls, Balance and Injury Research Centre, Neuroscience Research Australia, Sydney, NSW, Australia; ^3^University of New South Wales, Sydney, NSW, Australia; ^4^Department of Otolaryngology—Head and Neck Surgery, Johns Hopkins University School of Medicine, Baltimore, MD, United States; ^5^School of Biomedical Sciences and Pharmacy, University of Newcastle, Newcastle, NSW, Australia

**Keywords:** vestibulo-ocular reflex, peripheral vestibular disorders, expert panel assessment, community dwelling adults, benign paroxismal positional vertigo

## Abstract

People aged over 50 are the most likely to present to a physician for dizziness. It is important to identify the main cause of dizziness in order to develop the best treatment approach. Our goal was to determine the prevalence of benign paroxysmal positional vertigo (BPPV), and peripheral and central vestibular function in people that had experienced dizziness within the past year aged over 50. One hundred and ninety three community-dwelling participants aged 51–92 (68 ± 8.7 years; 117 females) were tested using the clinical and video head impulse test (cHIT and vHIT) to test high-frequency vestibular organ function; the head thrust dynamic visual acuity (htDVA) test to test high-frequency visual-stability; the dizziness handicap inventory (DHI) to measure the impact of dizziness; as well as sinusoidal and unidirectional rotational chair testing to test low- to mid-frequency peripheral and central vestibular function. From these assessments we computed the following measures: HIT gain; htDVA score; DHI score; sinusoidal (whole-body; 0.1–2 Hz with 30°/s peak-velocity) vestibulo-ocular reflex (VOR) gain and phase; transient (whole-body, 150°/s^2^ acceleration to 50°/s constant velocity) VOR gain and time constant; optokinetic nystagmus (OKN) gain and time constant (whole-body, 50°/s constant velocity rotation). Our study showed that BPPV, and peripheral or central vestibular hypofunction were present in 34% of participants, suggesting a vestibular cause to their dizziness. Over half (57%) of these with a likely vestibular cause had BPPV, which is more than twice the percentage reported in other dizzy clinic studies. Our findings suggest that the physical DHI score and VOR time constant were best at detecting those with non-BPPV vestibular loss, but should always be used in conjunction with cHIT or vHIT, and that the htDVA score and vHIT gain were best at detecting differences between ipsilesional and contralesional sides.

## Introduction

The vestibular system plays a key role in maintaining balance, stable gait, and stable vision during head movement via the vestibulo-ocular reflex (VOR). Vestibular disorders result in dizziness and imbalance, which contributes to an increased risk of falls and associated morbidity (1–4). Typically, injury to the vestibular system is localized to the peripheral vestibular organ and nerve, whereas aging is thought to affect the complete vestibular system including the vestibular nuclei of the brainstem with their commissural connections and the vestibulo cerebellum and associated pathways (5). Although neural changes due to aging have been observed within the vestibular system, it is not clear whether they directly correspond to behavioral changes in dizziness, imbalance, gait, falls, and visual instability during head movement (6, 7). For example, the prevalence of imbalance and dizziness in the elderly is ~30% (8); however, it is not clear whether that figure is mainly due to vestibular, psychological, muscular, or cardiovascular causes. The few studies that have examined the contributory causes of dizziness report contrasting findings [e.g., (9)]. Studies that have investigated the prevalence of vestibular impairment among people with dizziness have all been predominantly in a primary care setting and /or specialist ENT clinics; little data are available on the prevalence of vestibular causes of dizziness among an older community-dwelling population. This is an important issue to address because identifying the primary cause leads to targeted and effective rehabilitation, e.g., vestibular rehabilitation has been shown to improve the quality of life of patients suffering from imbalance and dizziness (10). Improvements in identifying these people are important so that they can receive timely rehabilitation treatment and potentially avoid further debilitating and life-threatening injuries.

We recently reported findings for 76 participants over the age of 50 who experienced significant dizziness within the past year (11)—the age group most likely to present to a physician with dizziness (1). That report revealed 38% of participants had a detectable peripheral vestibular disorder (29/76) and 1% a central vestibular disorder (1/76) that was the likely cause of their dizziness. Of those with a vestibular cause, 63% (19/30) had benign paroxysmal positional vertigo (BPPV), which was higher than previously reported—~25% in dizzy clinic populations (12, 13). Head thrust dynamic visual acuity (htDVA; 10) and sinusoidal (particularly 0.5–1 Hz) and transient VOR testing were identified as the most effective tests for detecting people with BPPV or vestibular hypofunction, whereas optokinetic nystagmus (OKN) testing and the dizziness handicap inventory (DHI) were only effective in detecting non-BPPV vestibular hypofunction (11).

A limitation of the Chau et al. (11) study was the small sample size. The present study builds on that work with data from an additional 117 participants. Here we aim to better: (i) identify the prevalence of BPPV, and peripheral and central vestibular hypofunction in people over the age of 50 who have experienced significant dizziness within the past year; (ii) compare performance in the DHI questionnaire and clinical tests of vestibular function between three vestibular status groups (vestibular “Lesion,” “BPPV,” or “Non-vestibular”) as decided by an expert panel and; (iii) determine the relationship between these test measures and age. For the tests that show a significant difference between Lesion and Non-vestibular group measures, thresholds are calculated and the sensitivity and specificity of the test are reported.

## Materials and Methods

### Participants

We studied 193 people with self-reported dizziness who had experienced at least one significant dizziness episode within the past year; 117 females and 76 males, aged 51–92 years old (mean = 68 ± 8.7). These were consecutive participants drawn from a larger sample of 305 people involved in a randomized-controlled trial of dizziness intervention (14). Recruitment methods and inclusion/exclusion criteria were identical to those we previously reported (11). In brief, participants were included if they experienced dizziness not currently treated (self-reported), lived independently in the community or a retirement village, and were aged at least 50 years. Participants were excluded if they experienced severe depressive symptoms or anxiety, a degenerative neurological condition, cognitive impairment, or a condition that required urgent treatment. All participants gave written and informed consent prior to participating in the study and the experimental protocol was approved by the Human Research Ethics Committee at the University of New South Wales.

### Testing Protocol

Participants first filled in a questionnaire about their dizziness history; it comprised a mix of dichotomous and open-ended questions about dizziness symptoms, the first time they experienced dizziness, most recent event, and the results of any medical investigations conducted. They then underwent tests in the following order: cardiovascular, clinical vestibular (eye examination, smooth pursuit, skew deviation, head-shaking nystagmus, clinical head impulse testing, Dix-Hallpike and roll test), walking trials, choice-stepping reaction time, physiological profile assessment, balance, and laboratory vestibular tests (video head impulse test, head thrust dynamic visual acuity, rotary chair testing). The present study focuses on presenting the results of the clinical and laboratory vestibular tests.

### Cardiovascular Assessment

Orthostatic hypotension was assessed with the tilt-table test (15). Orthostatic hypotension was defined as a reduction of 20 mmHg or more in systolic blood pressure or to ≤90 mmHg within 3 min of tilting (16). Delayed orthostatic hypotension was defined as a reduction of 20 mmHg or more in systolic blood pressure or of 10 mmHg or more in diastolic blood pressure after 3 min of upright tilt (17). Participants also undertook an electrocardiogram, which was reviewed by a medical doctor for the presence of any abnormality (e.g., arrhythmia) (16).

### Psychological Assessment

Anxiety was measured using the Generalized Anxiety Disorder 7 Item Scale (score ≥8 indicates clinically significant symptoms) (18) and depression using the Patient Health Questionnaire 9 Item Scale (score ≥10 indicates clinically significant symptoms) (19).

### Physiological Function, Balance, and Gait

Participants were assessed using the Short-Form Physiological Profile Assessment, which comprises five tests evaluating important functions of the human balance system: peripheral sensation, visual contrast sensitivity, lower limb strength, simple reaction time, and postural sway. Descriptions of the apparatus, procedures, and test–retest reliability for these tests are reported elsewhere (20). Participants also completed tests of: touch sensitivity at the lateral malleolus and first metatarsophalangeal joint using aesthesiometers (20) and controlled leaning balance using the coordinated stability test (21) in which a score ≥15 error points indicates impaired dynamic balance (22).

### Vestibular Testing

Vestibular testing took 2–3 h to complete for each participant. The last dizziness episode was categorized for each participant into six time periods: test day [prior to testing], last week, last month, last 3 months, last 6 months, or last year. The percentage (or ratio) of subjects within their group experiencing dizziness was calculated for each time period. Participants completed the Dizziness Handicap Inventory (DHI) (23), followed by Dix-Hallpike and roll testing to detect BBPV (24), the clinical head impulse test (cHIT) was deemed positive when the abnormal presence of corrective saccades was detected by the operator during the vestibulo-ocular reflex (VOR) head impulse response, and was negative otherwise. The video head impulse test (vHIT) was used to measure the VOR gain (eye/head velocity) during head impulses (high-frequency i.e., 2–3 Hz), and dynamic visual acuity testing was used to measure visual acuity during head impulses in all three semicircular canal planes (also known as head thrusts, i.e., htDVA). To help with data interpretation htDVA scores were classified as Normal [≤ 0.158 LogMAR, i.e., ≤2 SDs from normal mean, see (25)], Borderline (>0.158 and ≤0.316, 2–4 SDs), and Abnormal (> 0.316). In addition, rotary chair testing was performed to measure the low- to mid-frequency (0.1–2 Hz) VOR and visual VOR (VVOR) gains and phases. Rotary chair testing was also used to measure the transient VOR (in response to an acceleration step), optokinetic nystgamus (OKN) gains and time constants. All test methods and processing are identical to those described in Chau et al. (11). The only additional test included in this study was vHIT with head impulses in the horizontal canal plane, which was performed in the last 87 participants.

### Video Head Impulse Test (vHIT)

Angular head velocity and left eye rotations were measured using the EyeSeeCam video-oculography goggle system (Denmark) using the same methods as previously described in detail (26, 27). In brief, the EyeSeeCam system measures head velocity and eye position at a sample rate of 220 Hz. The digital video camera, infra-red mirror, and inertial measurement unit are rigidly mounted onto a lightweight swim goggle frame securely placed on the subject's head. Subjects were asked to fixate visual targets at known angles to calibrate horizontal and vertical eye position. After differentiating calibrated eye position, head and eye velocity data were digitally filtered with a 50-tap zero-phase low pass FIR filter with a bandwidth of 50 Hz.

The passive head impulse test was used to measure leftward and rightward head rotation VOR responses (28). A head impulse consists of a horizontal, unidirectional (leftward or rightward in randomized order) head rotation with ~200 ms duration, ~10° peak amplitude, ~150°/s peak velocity, and ~3,000°/s^2^ peak acceleration. The onset of each head impulse was determined by fitting the magnitude of horizontal head velocity to a polynomial curve vs. time. The impulse onset was defined as the time where the magnitude of the fitted curve was >2% of the curve's peak magnitude. Head impulses with peak magnitude lower than 150°/s or >300°/s were removed from the analysis. Eye (and corresponding head) traces with blinks or other artifacts were also not included. The VOR gain at each sample point during the 30 ms period immediately prior to peak head impulse velocity (corresponding to 6–7 gain values at 220 Hz sample rate) was calculated as the magnitude of eye velocity divided by head velocity. We reported the VOR gain as the median of those gains (27).

### Participant Rehabilitation Categorization

A geriatrician, vestibular physiotherapist, vestibular scientist, and psychologist together evaluated each participant's medical history and performance on a range of psychological questionnaires as well as physiological tests including the vestibular tests outlined above (14). Each participant was allocated to a group depending on the likely cause of dizziness: vestibular hypofunction, which would make them suitable for vestibular rehabilitation exercises (vestibular “Lesion” group); BPPV, which would make them suitable for Epley maneuver treatment (“BPPV” group); and those with non-vestibular cause (“Non-vestibular” group).

### Statistical Analysis

Statistical analysis was performed using SPSS version 23 (IBM, USA) and Excel 2013 (Microsoft, USA) software. A mixed-design analysis of variance (ANOVA) with two-, three-, and four-factor interactions was used to analyze the data (29). For sinusoidal VOR gain and phase analysis the independent ANOVA variables were: test frequency (*frequency*: 0.1, 0.2, 0.4, 0.5, 0.8, 1, 1.6, and 2 Hz), test type (*test*: “VVOR” or “VOR”), and participant vestibular rehabilitation group (*group*: “Lesion,” “Non-vestibular,” or “BPPV”). For transient VOR gain and time constant analysis, the independent variables were: vestibular stimulus type (*stimulus*: “excitatory,” “inhibitory”) and how it was applied with respect to the lesion side (*same side*: “yes” or “no”) and “*group*,” whereas for OKN gain and time constant analysis the variables were: “*same side*” and “*group*.” For htDVA score analysis the variables were: canal (*canal*: “horizontal,” “anterior,” and “posterior”), “*same side*” and “*group*,” whereas for total physical, total emotional, total functional, and grand total DHI scores the only variable was “*group*.” Participant *age* was a covariate in all ANOVAs. Variables with ≥95% confidence were reported as 5% significant, and those with 90–95% confidence as close to 5% significance trends. Effect size η^2^ for ANOVA, was reported as Small 0.005–0.05, Medium 0.05–0.125, or Large >0.125; and effect size *cohen-d* for *z-test* (to compare proportions), was reported as Small 0.15–0.45, Medium 0.45–0.75, or Large > 0.75 (30). The correlation co-efficient was also calculated between test measures and reported as the Pearson product-moment correlation r with a value between −1 to +1, with |r| closer to 1 indicating higher correlation.

## Results

### Demographics

Of the 193 participants, 126 (68 female, 58 male) were allocated to the Non-vestibular group, 39 (32 female, seven male) to the BPPV group, and 28 (17 female, 11 male) to the Lesion group. There was no difference in mean (±SD) ages between the Non-vestibular (67.6 ± 8.8 years), BPPV (69.8 ± 8.2 years), and Lesion (66.8 ± 9.3 years) groups (ANOVA: “*group*” variable, *P* = 0.946, η^2^ = 0.001).

For the Non-vestibular group, 25 participants experienced a dizzy episode on the day of testing (prior to testing), 41 in the last week, 33 in the last month, 13 in the last 3 months, nine in the last 6 months, and one in the last year. For the BPPV group, 12 on the day, 14 in the last week, six in the last month, three in the last 3 months, and four in the last 6 months. For the Lesion group, six on the day, eight in the last week, six in the last month, three in the past 3 months, two in the last 6 months, and two in the past year. The maximum difference in percentage between two groups across all time periods was on the day of testing between the Non-vestibular (25/126 = 19.8%) and BBPV (12/39 = 30.8%) groups. However, this 11% difference was not significant (*z-test*: *P* = 0.157, *cohen-d* = 0.111), suggesting that there was no difference between the distribution of dizziness episodes over time between groups.

Dix-Hallpike testing in the 39 participants with BPPV revealed: 9 had upbeat left torsional nystagmus during left posterior canal testing only; 16 had upbeat right torsional nystagmus during right posterior canal testing only; 10 had upbeat left torsional nystagmus during left posterior canal testing and upbeat right torsional nystagmus during right posterior canal testing; three had horizontal nystagmus during right horizontal canal testing; and one had persistent horizontal nystagmus during left horizontal canal testing suggesting cupuloliathisis. Nystagmus did not persist in 38/39 participants with BPPV suggesting canalithiasis. Head movement was a clear trigger for dizziness (spinning sensation) for 30/39 participants with BPPV.

Of the 126 participants classified in the Non-vestibular group, 49 did not exhibit any abnormal results in any of the assessments. Of the remaining 77 participants, nearly half of them exhibited multiple dizziness-related deficits/factors as reflected by the therapies recommended for them [see (14)]. Regarding psychiatric etiologies, 12 participants showed clinically significant symptoms of anxiety (Generalized Anxiety Disorder 7 item (GAD-7) scale score >7) and 14 showed clinically significant symptoms of depression (Patient Health Questionnaire 9 item (PHQ-9) score >9). Regarding cardiovascular problems, 21 participants exhibited orthostatic hypotension on the tilt table test, and 8 exhibited delayed orthostatic hypotension. Three participants had low blood pressure and six had an abnormal electrocardiogram. Twenty participants had poor balance whereby they were unable to stand for 30 s on a foam mat with eyes closed (postural sway on foam eyes closed) and/or had poor leaning and coordinated balance (coordinated stability score ≥15 points). Seven participants had poor touch sense on the feet (lateral malleolus/1st metatarsophalangeal joint filament ≥6) and /or knee position sense (error in foot matching task >5 degrees). Two participants had suspected vestibular migraines based on history.

Of the 28 participants classified in the Lesion group, one had a history of idiopathic labyrinth failure diagnosed from an MRI, two had suspected history of Mal de Debarquement, two had suspected Meniere's disease, five had history of hypofunction from suspected viral origin, three had nil findings from specialist investigations (ENT, Neurologist, negative MRI), four had no specific diagnosis established from specialist vestibular investigations (ENT, Neurologist, negative MRI), one was prescribed antidepressants by a neurologist, seven had no history of investigations beyond consulting a GP, and three had a history of migraines without any further investigations. Half of the participants (*n* = 14) reported tinnitus and /or aural fullness.

### Clinical Head Impulse Test (cHIT)

**Figure 1:**
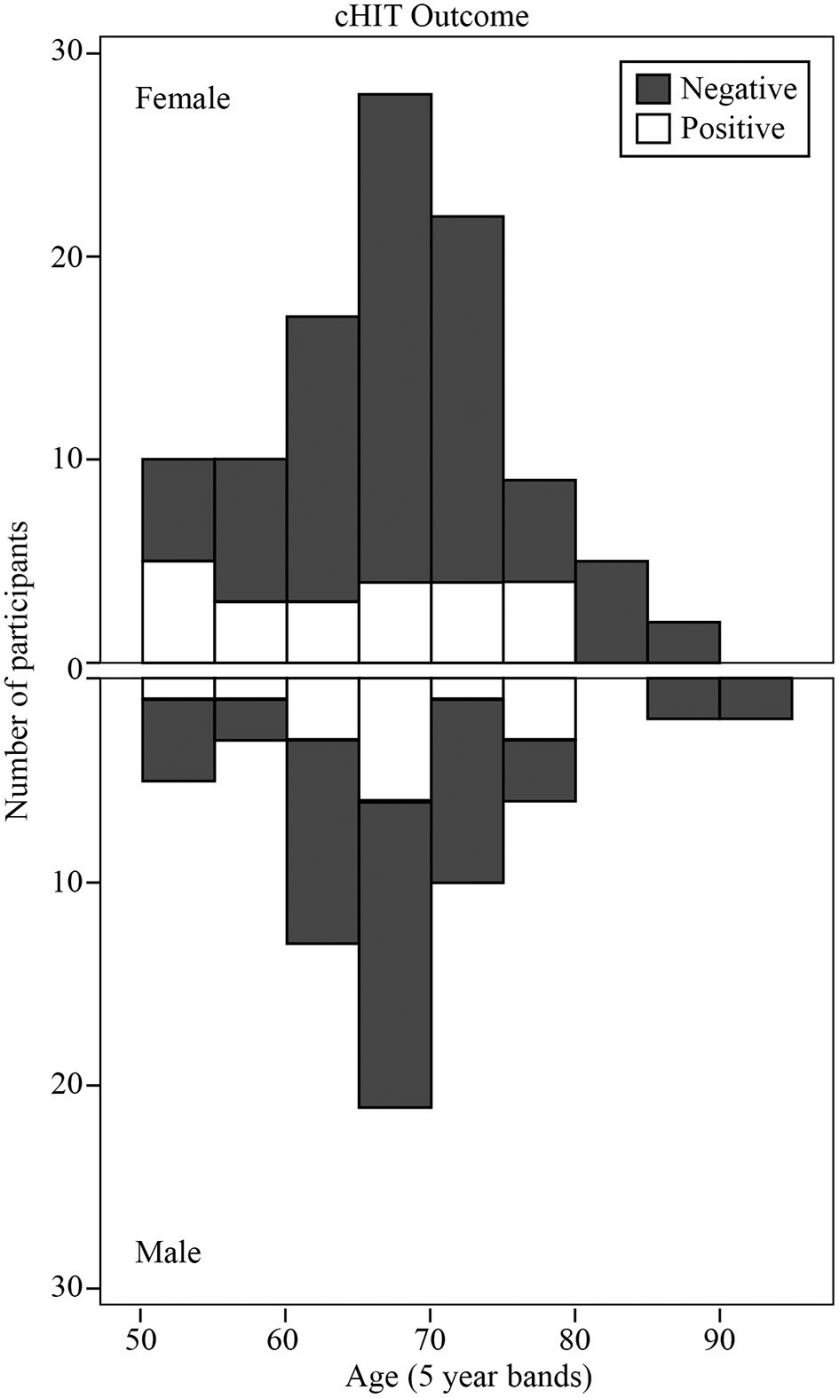
Histogram of clinical Head Impulse Test (cHIT) results binned as Positive or Negative vs. age (with 5-years bands).

cHIT was performed in 165/193 participants. Muscle stiffness or pain when moving their head preventing the other 28 from being tested. Figure 1 shows the histogram of cHIT results binned as Positive or Negative vs. age (with 5-years bands). The proportion of participants with positive cHIT decreased with age. Linear regression analysis revealed that the proportion decreased by 0.04 every 5 years after age 50 from 0.42 (*R*^2^ = 0.44; constant = 0.416, t = 3.94, *P* < 0.01, slope co-efficient = −0.044, *t* = −2.34, *P* < 0.05). The cHIT was positive in 17/106 participants in the Non-vestibular group, 11/32 participants in the BPPV group, and 10/27 participants in the Lesion group. There were significant differences in these proportions between the Non-vestibular and Lesion groups (*z-test*: *P* < 0.02, *cohen-d* = 0.210), and Non-vestibular and BPPV groups (*z-test*: *P* < 0.05, *cohen-d* = 0.192), but not between the Lesion and BPPV groups (*z-test*: *P* = 0.834, *cohen-d* = 0.026). cHIT sensitivity (true positives) was 0.37 (10/27) and specificity (true negatives) was 0.84 (89/106).

### Video Head Impulse Testing (vHIT)

**Figure 2:**
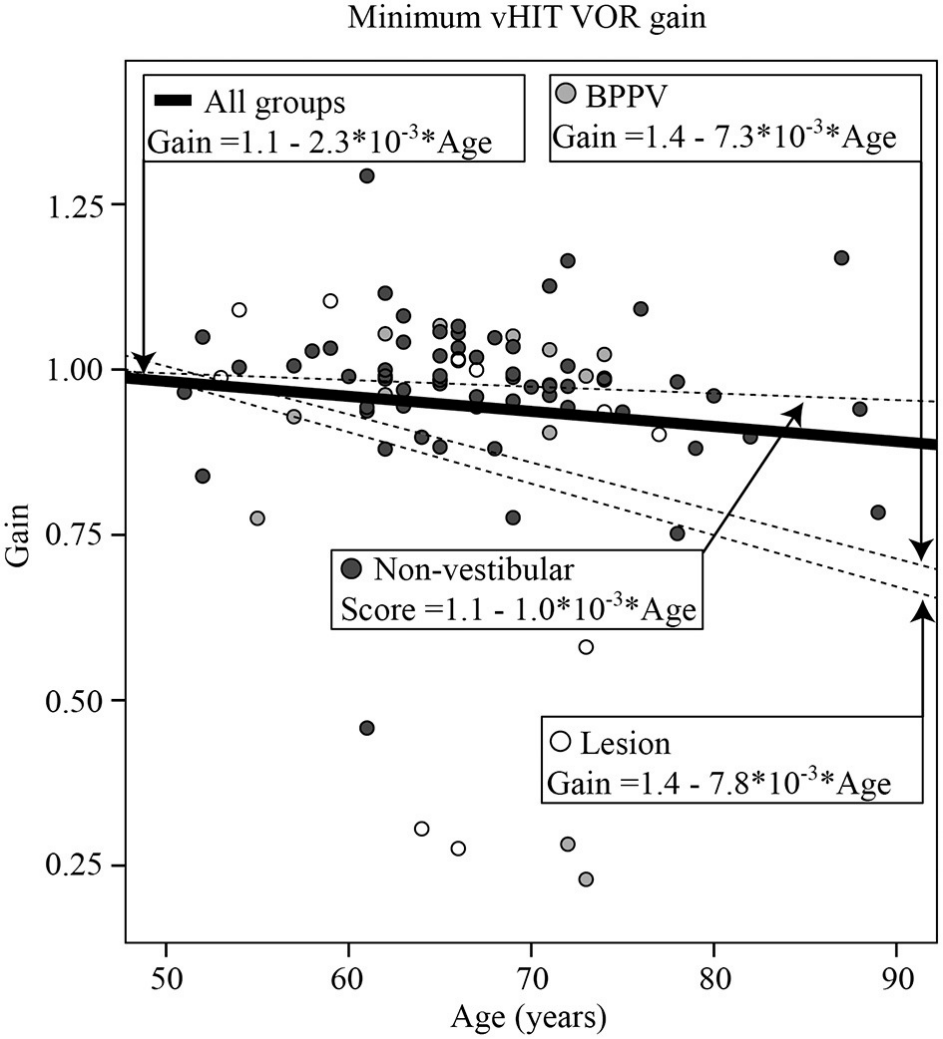
Minimum (between leftward and rightward head rotations) video Head Impulse Test (vHIT) vestibulo-ocular reflex (VOR) gain calculated for each participant vs. their age, clustered by group with group and overall line-fits.

The vHIT was performed in the last 87 participants (13 Lesion, 14 BPPV, and 60 Non-vestibular). There was a significance difference in vHIT gain between ipsilesional and contralesional head rotations [ANOVA: *same side, F*_(1, 289)_ = 5.74, *P* < 0.02, η^2^ = 0.02]. The mean gains toward the ipsilesional and contralesional sides were, respectively 0.92 ± 0.26 and 1.03 ± 0.12. Figure 2 shows the minimum (between leftward and rightward) vHIT VOR gain calculated for each participant vs. their age, clustered by group with group and overall line-fits. There was no significant difference between group line-fits [ANOVA: *group, F*_(2, 86)_ =0.34, *P* = 0.72, η^2^ = 0.08; *group* * *age covariate, F*_(2, 86)_ = 0.62, *P* = 0.54, η^2^ = 0.015]. Similarly, age did not significantly affect the vHIT gain [ANOVA: *age covariate, F*_(1, 86)_ = 2.22, *P* = 0.14, η^2^ = 0.027]. Out of the six gains ≤ 0.75, 5 (83%) were ipsilesional (for 3 Lesion and 2 BPPV group participants), whereas eight out of the nine gains between 0.75 and 0.90 were contralesional, suggesting that a gain threshold of 0.75 was optimal for peripheral organ lesion detection for these participants. Using the minimum vHIT gain (per subject) and 0.75 as the threshold vHIT, the sensitivity and specificity were, respectively, 0.23 (3/13) and 0.98 (59/60).

### Head Thrust Dynamic Visual Acuity (htDVA)

**Figure 3:**
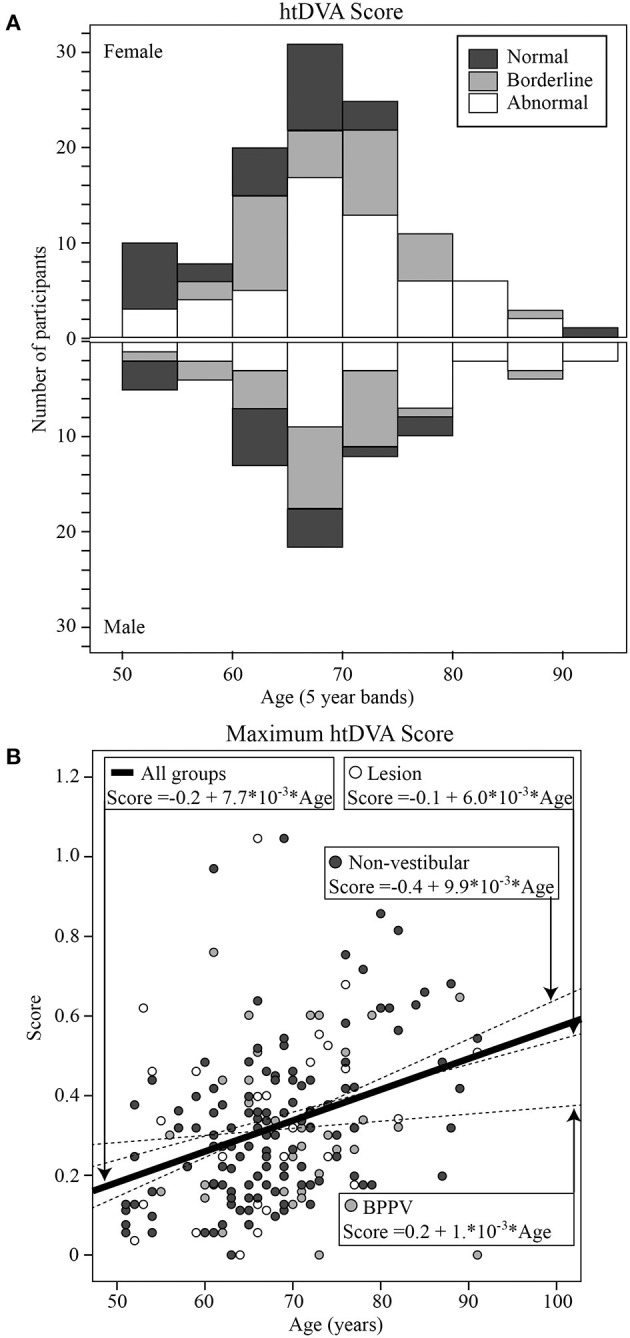
**(A)** Histogram of htDVA scores binned as normal, borderline, and abnormal vs. age (with 5-years bands). **(B)** Maximum htDVA score [across all canal planes [left anterior, left posterior, left horizontal, right anterior, right posterior, right horizontal]] calculated for each participant vs. their age, clustered by group with group and overall line-fits.

Mean htDVA scores for Non-vestibular, BPPV, and Lesion groups as well as the proportion of participants classified as Normal, Borderline, and Abnormal are shown in Table 1. The factors that affected the htDVA score were age [ANOVA: *age covariate, F*_(1, 942)_ = 155.72, *P* < 0.001, η^2^ = 0.144], canal tested [ANOVA: *canal, F*_(2, 942)_ = 6.45, *P* < 0.002, η^2^ = 0.014], participant group [ANOVA: *group, F*_(2, 942)_ = 6.23, *P* < 0.002, η^2^ = 0.013], and whether the head rotation was toward the lesion side (for the Non-vestibular group left and right head rotations were pooled and considered contralesional rotations) [ANOVA: *same side, F*_(1, 942)_ = 8.81, *P* < 0.005, η^2^ = 0.009]. Linear regression analysis revealed that htDVA score increased by 0.007 per year after age 50 from 0.083 (*R*^2^ = 0.138; constant = −0.267, t = −6.75, *P* < 0.001, slope co-efficient = 0.007, t = 12.28, *P* < 0.001). Figure 3A shows the histogram of htDVA scores binned as Normal, Borderline, and Abnormal vs. age (with 5-years bands). The proportion of participants with an Abnormal score in participants aged 65 and above was more than double compared to those aged below 65. Figure 3B shows the maximum htDVA score (across all canal planes) calculated for each participant vs. their age, clustered by group with group and overall line-fits. Age significantly affected the maximum htDVA score [ANOVA: *age covariate, F*_(1, 189)_ = 9.43, *P* < 0.005, η^2^ = 0.049]. Using the maximum htDVA score (per subject) and 0.316 as the threshold htDVA, sensitivity and specificity were, respectively 0.54 (15/28) and 0.52 (64/124). There was also a significant difference between BPPV and Non-vestibular group line-fits [ANOVA: *group, F*_(2, 161)_ = 3.56, *P* = 0.06, η^2^ = 0.022; *group* * *age covariate, F*_(2, 161)_ = 3.92, *P* < 0.05, η^2^ = 0.024]. Between the BPPV and Non-vestibular groups, anterior canal htDVA scores were significantly different, and posterior canal scores were close to 5% significantly different [Anterior canal: ANOVA *group, F*_(1, 242)_ = 5.78, *P* < 0.02, η^2^ = 0.024; Posterior canal: ANOVA *group, F*_(1, 228)_ = 3.2, *P* = 0.070, η^2^ = 0.015].

**Table 1:** Summary of mean htDVA scores for Non-vestibular, Lesion (Non-BPPV), and BPPV groups as well as the proportion of participants classified as Normal, Borderline, and Abnormal (≤0.158, >0.158, and ≤0.316, >0.316 logMAR, respectively).

**Side**	**Canal**	**Non-vestibular**			**Lesion (Non-BPPV)**			**BPPV**		
		**Affected**	**No**.	**Mean ± SD**	**Affected**	**No**.	**Mean ± SD**	**Affected**	**No**.	**Mean ± SD**
Ipsi.	Hor.	Normal	67/124	0.202 ± 0.151	Normal	14/35	0.262 ± 0.210	Normal	25/46	0.208 ± 0.146
		Borderline	35/124		Borderline	9/35		Borderline	15/46	
		Abnormal	22/124		Abnormal	12/35		Abnormal	6/46	
	Ant.	Normal	55/94	0.199 ± 0.177	Normal	15/32	0.230 ± 0.172	Normal	20/34	0.166 ± 0.100
		Borderline	24/94		Borderline	5/32		Borderline	12/34	
		Abnormal	15/94		Abnormal	12/32		Abnormal	2/34	
	Post.	Normal	35/89	0.228 ± 0.149	Normal	12/32	0.282 ± 0.216	Normal	7/32	0.265 ± 0.138
		Borderline	33/89		Borderline	9/32		Borderline	17/32	
		Abnormal	21/89		Abnormal	11/32		Abnormal	8/32	
Cont.	Hor.	Normal	73/122	0.196 ± 0.159	Normal	15/19	0.157 ± 0.120	Normal	16/26	0.207 ± 0.173
		Borderline	30/122		Borderline	2/19		Borderline	5/26	
		Abnormal	19/122		Abnormal	2/19		Abnormal	5/26	
	Ant.	Normal	42/93	0.229 ± 0.173	Normal	11/18	0.151 ± 0.091	Normal	14/21	0.145 ± 0.079
		Borderline	28/93		Borderline	5/18		Borderline	7/21	
		Abnormal	23/93		Abnormal	2/18		Abnormal	0/21	
	Post.	Normal	35/88	0.253 ± 0.173	Normal	7/18	0.198 ± 0.146	Normal	10/19	0.201 ± 0.112
		Borderline	29/88		Borderline	8/18		Borderline	6/19	
		Abnormal	24/88		Abnormal	3/18		Abnormal	3/19	

*For the Non-vestibular group, ipsilesional is left and contralesional is right*.

### Dizziness Handicap Inventory (DHI)

**Figure 4:**
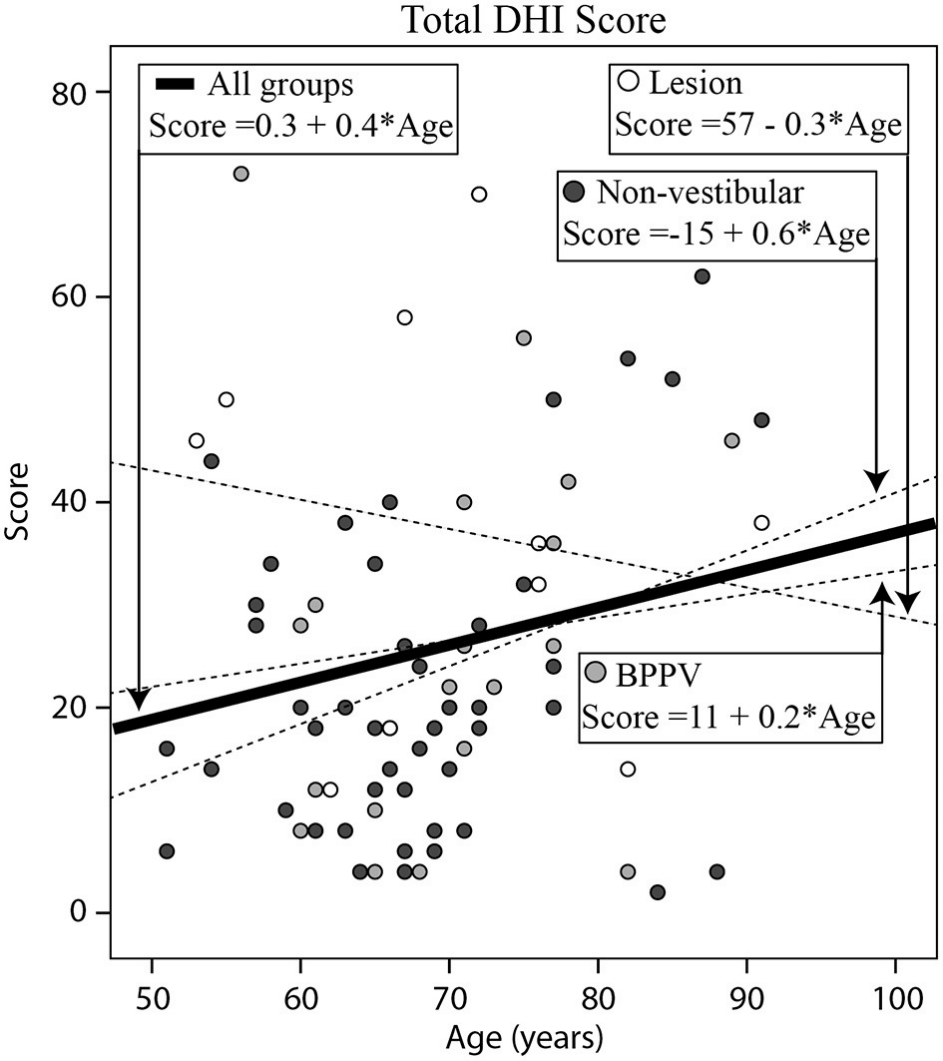
Histogram of total (Emotional + Functional + Physical) Dizziness Handicap Inventory (DHI) score for each participant vs. their age, clustered by group with group and overall line-fits.

Figure 4 shows the total DHI score for each participant vs. their age, clustered by group with group and overall line-fits. There was no significant difference between group line-fits [ANOVA: *group, F*_(2, 75)_ = 1.78, *P* = 0.18, η^2^ = 0.048; *group* * *age covariate, F*_(2, 75)_ =1.21, *P* = 0.30, η^2^ = 0.033]. Similarly, age did not significantly affect the total DHI score [ANOVA: *age covariate, F*_(1, 75)_ = 0.48, *P* = 0.49, η^2^ = 0.007]. The mean total scores for the Lesion, BPPV, and Non-vestibular groups were respectively, 30.1 ± 18.4, 28.9 ± 17.4, and 23.8 ± 16.9. There was also a significant difference in the physical sub-score between groups [ANOVA: *group, F*_(2, 190)_ = 4.65, *P* < 0.05, η^2^ = 0.047], but not the emotional [ANOVA: *group, F*_(2, 190)_ = 1.59, *P* = 0.21, η^2^ = 0.017] and functional [ANOVA: *group, F*_(2, 190)_= 1.29, *P* = 0.278, η^2^ = 0.014] sub-scores derived from the inventory. The mean physical scores for the Lesion, BPPV, and Non-vestibular groups were, respectively, 8.5 ± 8.4, 6.6 ± 6.6, and 5.9 ± 6.4. Using the physical score and 5.9 as the threshold, DHI sensitivity and specificity were, respectively, 0.80 (8/10) and 0.21 (10/47).

### Sinusoidal Horizontal VOR and VVOR Testing

**Figure 5:**
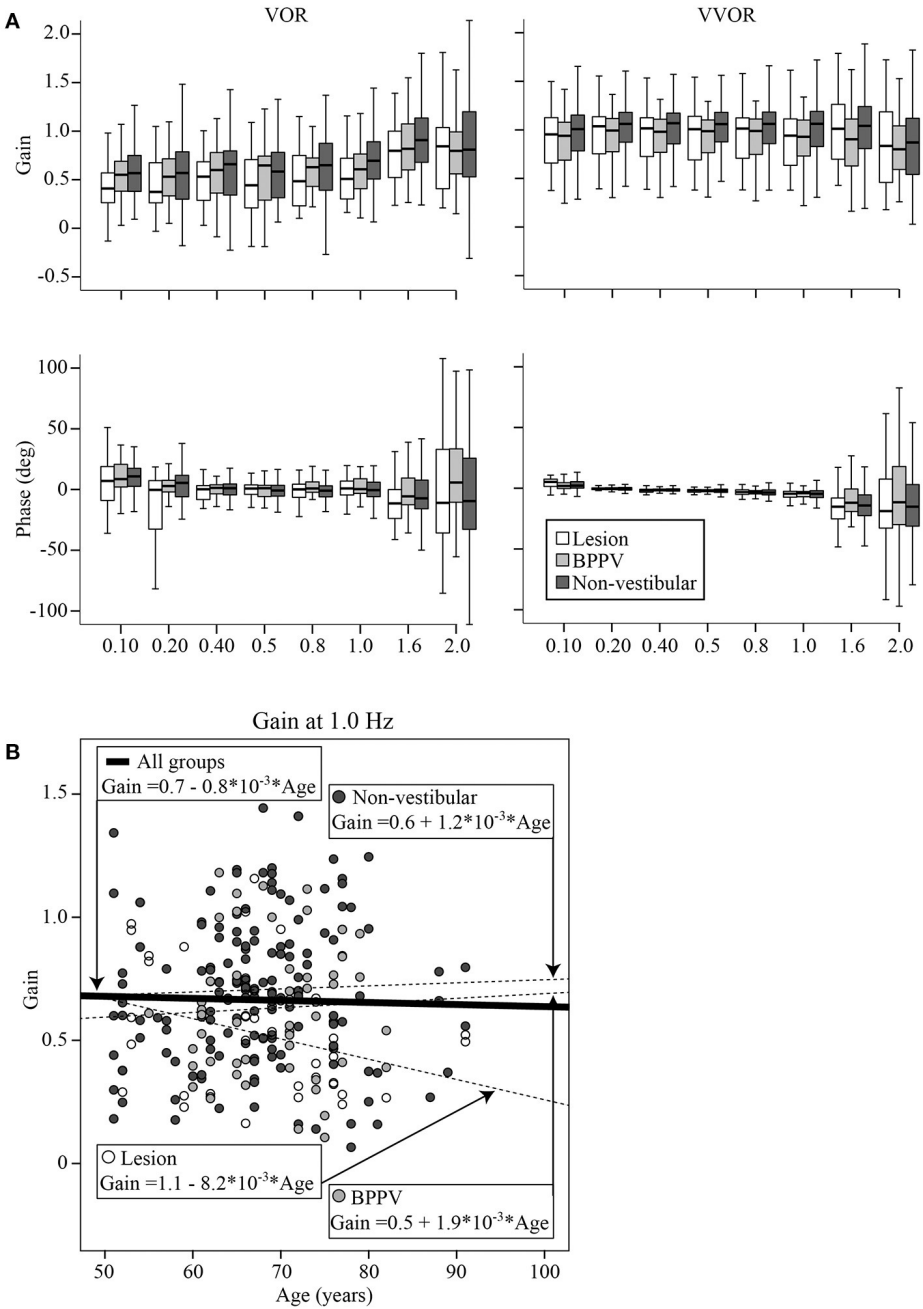
**(A)** Boxplots of the VOR (left column) and VVOR (right column) gains (top row) and phases (bottom row) across test frequencies for each group (Lesion group in white, BPPV in light gray, and Non-vestibular in dark gray). Each box begins at the first quartile, ends at the third quartile, denotes the median with a horizontal stripe and has whiskers denoting the maximum and minimum values. **(B)** VOR gain at 1 Hz for each participant vs. their age, clustered by group with group and overall line-fits.

Figure 5A shows boxplots of the VOR (left column) and VVOR (right column) gains (top row) and phases (bottom row) across test frequencies for each group (Lesion group in white, BPPV in light gray, and Non-vestibular in dark gray). Each box shows the median and goes from the first to the third quartile with whiskers denoting the minimum and maximum values.

The factors that significantly affected the gain were the test protocol [VOR or VVOR; ANOVA: *test, F*_(1, 4, 288)_ = 540.40, *P* < 0.0001, η^2^ = 0.114], participant group [ANOVA: *F*_(2, 4, 288)_ = 25.42, *P* < 0.0001, η^2^ = 0.012], test frequency [ANOVA: *F*_(7, 4, 288)_ = 12.71, *P* < 0.0001, η^2^ = 0.021], and age [ANOVA: *age covariate, F*_(1, 4, 288)_ = 7.57, *P* < 0.01, η^2^ = 0.002]. There was a significant interaction between test protocol and frequency [ANOVA: *F*_(7, 4, 288)_ = 15.73, *P* < 0.0001, η^2^ = 0.026], suggesting that frequency most affected gains during VOR testing. There was also a significant interaction between test protocol and participant group (ANOVA: *P* < 0.05, η^2^ = 0.002), suggesting that gain differences between groups were most evident during VOR testing. The mean VVOR gain across all conditions was close to unity at 0.97 ± 0.36, whereas the mean VOR gain was about ~33% lower at 0.63 ± 0.39. The mean VOR gains across conditions for the Lesion, BPPV, and Non-vestibular groups were, respectively, 0.55 ± 0.40, 0.60 ± 0.33, and 0.67 ± 0.40. The gain spread between groups was largest at 1 Hz where the mean VOR gain for the Lesion, BPPV, and Non-vestibular groups were, respectively, 0.53 ± 0.30, 0.63 ± 0.28, and 0.71 ± 0.30. Linear regression analysis revealed that the relationships between VVOR or VOR gain with age were poor linear fits with respective *R*^2^ values of 0.003 and 0.001. Figure 5B shows the VOR gain at 1 Hz for each participant vs. their age, clustered by group with group and overall line-fits. There was no significant difference between group line-fits [ANOVA: *group, F*_(2, 253)_ = 0.94, *P* = 0.39, η^2^ = 0.008; *group* * *age covariate, F*_(2, 253)_ = 1.66, *P* = 0.19, η^2^ = 0.013]. Similarly, age did not significantly affect the VOR gain at 1 Hz [ANOVA: *age covariate, F*_(1, 253)_ =0.38, *P* = 0.54, η^2^ = 0.002]. Using the VOR gain at 1 Hz and 0.75 as the threshold, sinusoidal testing sensitivity and specificity were, respectively, 0.59 (32/54) and 0.46 (73/157).

Phase followed a similar pattern to gain. The factors that affected phase were test protocol [VOR or VVOR; ANOVA: *test, F*_(1, 4,348)_ = 11.59, *P* < 0.001, η^2^ = 0.003], participant group [ANOVA: *F*_(2, 4,348)_ = 5.44, *P* < 0.005, η^2^ = 0.003], test frequency [ANOVA: *F*_(7, 4,348)_ = 9.49, *P* < 0.0001, η^2^ = 0.015], and age [ANOVA: *age covariate, F*_(1, 4,348)_ = 12.57, *P* < 0.0001, η^2^ = 0.003]. There was a significant interaction between test protocol and participant group [ANOVA: *F*_(2, 4,348)_ = 3.84, *P* < 0.05, η^2^ = 0.002], suggesting that phase differences between groups were most evident during VOR testing. The VVOR phase decreased (lags) with frequency by 6.8° per Hz starting at 1.3° at 0.1 Hz (*R*^2^ = 0.076; constant = 2.014, t = 4.13, *P* < 0.001, slope co-efficient = −6.832, t = −13.73, *P* < 0.001). Whereas, VOR phase with frequency was a poor linear fit (*R*^2^ = 0.001). The mean VOR phase across conditions for the Lesion, BPPV, and Non-vestibular groups were, respectively, −4.63 ± 31.70°, 2.09 ± 24.08°, and −1.07 ± 28.56°. The phase spread between groups was largest at 1.6 Hz where the mean VOR phase for the Lesion, BPPV, and Non-vestibular groups were, respectively, −12.42 ± 34.47°, 2.41 ± 26.75°, and 0.1 ± 34.76°. Linear regression analysis revealed that the relationship between VVOR or VOR phase with age were poor linear fits with respective *R*^2^ values of 0.005 and 0.003.

### Transient (Acceleration Steps) Horizontal VOR Testing

**Figure 6:**
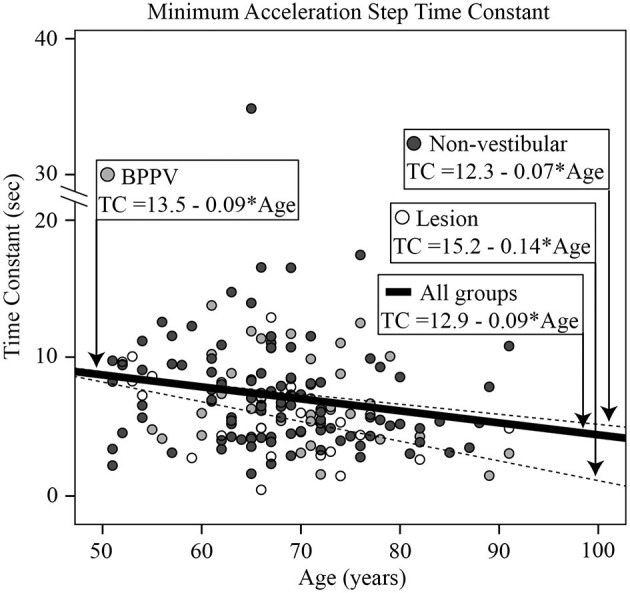
Histogram of the minimum acceleration step time constant [across all directions and stimuli [left excitatory, left inhibitory, right excitatory, right inhibitory]] for each participant vs. their age, clustered by group with group and overall line-fits.

The factors which affected the acceleration step time constant were age [ANOVA: *age covariant, F*_(1, 601)_ = 20.46, *P* < 0.0001, η^2^ = 0.034] and group [ANOVA: *group, F*_(2, 601)_ = 3.03, *P* < 0.05, η^2^ = 0.010]. The mean time constant across conditions for the Lesion, BPPV, and Non-vestibular groups were, respectively, 7.9 ± 4.1, 9.4 ± 3.8, and 9.6 ± 5.4 s. Linear regression revealed that the time constant decreased with age by 0.1 s per year starting at 11.1 s at age 50 (*R*^2^ = 0.032; constant = 16.03, t = 10.51, *P* < 0.001, slope co-efficient =-0.099, t = =-4.46, *P* < 0.001). Figure 6 shows the minimum time constant (between leftward and rightward, inhibitory and excitatory) for each participant vs. their age, clustered by group with group and overall line-fits. Age significantly affected the time constant [ANOVA: *age covariate, F*_(1, 162)_ = 5.94, *P* < 0.02, η^2^ = 0.036]. However, there was no significant difference between group line-fits [ANOVA: *group, F*_(2, 162)_ = 0.10, *P* = 0.90, η^2^ = 0.001; *group* * *age covariate, F*_(2, 162)_ = 0.27, *P* = 0.77, η^2^ = 0.003]. Using the minimum time constant (per subject) and 9.6 as the threshold time constant, sensitivity and specificity were, respectively, 0.88 (21/24) and 0.20 (21/104). The only factor that affected the acceleration step gain was whether the stimulus was excitatory or inhibitory [ANOVA: *stimulus, F*_(1, 597)_ = 6.24, *P* < 0.02, η^2^ = 0.011]. The mean acceleration step gain toward the excitatory and inhibitory sides were, respectively, 0.86 ± 0.24 and 0.79 ± 0.23. The excitatory response of one participant revealed an exponential decay longer than the normal duration as previously reported (11).

### Optokinetic Testing (OKN)

There was a close to 5% significance difference in OKN time constant between ipsilesional and contralesional whole-body rotations (ANOVA: *same side, F*_(1, 242)_ = 2.96, *P* = 0.087, η^2^ = 0.012]. The mean time constants toward the ipsilesional and contralesional sides were, respectively, 2.7 ± 2.0 and 3.8 ± 3.4 s. The only factor to affect the OKN gain was age [ANOVA: *age covariant F*_(1, 240)_ = 4.32, *P* < 0.05, η^2^ = 0.018]. Linear regression revealed that the gain decreased with age by 0.004 per year starting at 0.683 at age 50 (*R*^2^ = 0.017; constant = 0.883, t = 7.05, *P* < 0.001, slope co-efficient = −0.004, t = −2.04, *P* < 0.05). The mean OKN gain across all conditions and groups was 0.63 ± 0.25.

### Correlation Between Measures

Pairwise correlations between all the tests described above were performed: four of these were statistically significant. The maximum htDVA score significantly correlated with the minimum vHIT gain (Pearson correlation r = −0.343, *P* < 0.005). This correlation increased when only the Lesion group data were included in the analysis (Pearson correlation r = −0.847, *P* < 0.001). Similarly, there was a significant correlation between the VOR gain measured at 1 Hz and the minimum vHIT gain (Pearson correlation r = 0.461, *P* < 0.001), which also increased when only the Lesion group data were included in the analysis (Pearson correlation r = 0.838, *P* < 0.001). The acceleration step time constant significantly correlated with minimum vHIT (Pearson correlation r = 0.310, *P* < 0.02) and the VOR gain at 1 Hz (Pearson correlation r = 0.231, *P* < 0.01).

## Discussion

Thirty four percent of participants had a detectable peripheral vestibular disorder (65/193) and 0.5% had a central vestibular disorder (1/193), which was the likely cause of their dizziness. It is likely that this percentage is a lower limit and actually larger than measured due to failure in detecting transient vestibular losses, e.g., BPPV that resolved before the day of testing. These findings are consistent with our previous report in 76 subjects and suggest about one third of people aged over 50 years with symptoms of dizziness have a vestibular cause (11). Of those with a vestibular cause, 57% (37/65) had BPPV. The prevalence of BPPV in this community sample is more than twice the previously reported ~25% in dizziness clinic patients (12, 13). Interestingly, Maarsingh et al. (9) reported BPPV in only 14% of 417 primary care patients with persistent dizziness. This large difference in BPPV rate (14 vs. 57%) might be due to the different combination of tests performed (Otoscopy, Dix-Hallpike and Roll testing, and Audiometry), setting (primary care vs. community-dwelling), dizziness complaint (persistent dizziness vs. dizziness in the past year), and difference in ages (mean age 78 vs. 68 in the present study).

### Head Thrust Dynamic Visual Acuity (htDVA)

For the Non-vestibular group, htDVA scores were similar between left and right sides (both counted as contralesional), and there was a significant difference between ipsilesional and contralesional sides. However, the sensitivity and specificity at detecting those in the Lesion group was moderate at ~0.5. The BPPV group had significantly worse anterior canal htDVA scores, and close to 5% significantly worse posterior canal scores compared to the Non-vestibular group, suggesting that BPPV could be affecting the VOR gain. We did not measure vertical canal VOR gains, but one explanation could be that because BPPV results in an inappropriate eye movement during head rotations in the plane of the vertical canals, the vertical canal gain is driven down. There is some evidence, albeit inconclusive, suggesting that the posterior canal VOR gain is reduced in patients with posterior canal BPPV (31). A reduction in VOR gain would result in the perseverance of eye movements with smaller magnitude, which presumably would reduce the sensation of vertigo, but at the cost of visual stability, i.e., a decrease in visual acuity during head movements. Maximum htDVA score increased with age. In fact, the proportion of participants with an Abnormal score in participants aged 65 and above was more than double compared to those aged below 65. Maximum htDVA score correlated with the minimum VOR gain measured, suggesting a relationship between high-frequency VOR gain and high-frequency visual acuity.

### Dizziness Handicap Inventory Scores (DHI)

Total DHI was not useful in discriminating between participants with vestibular and non-vestibular causes. The best DHI indicator was the total physical score, which was significantly different between the Lesion and BPPV/Non-vestibular (combined) groups. Using the physical score the DHI was highly sensitive at detecting those in the Lesion group, but had poor specificity. Participants with BBPV perceived their physical handicap to be somewhere between Lesion and Non-vestibular participants, however, the difference in score between the Lesion and Non-vestibular groups was only 2.6 points. There was no relationship between DHI score and age.

### Sinusoidal Horizontal VOR and VVOR

As in the prior smaller study (11), sinusoidal VOR gain was the best indicator of the cause of dizziness. However, unlike the prior study the sinusoidal frequency for optimal detection was 1 Hz. At 1 Hz the VOR gain in the Non-vestibular group was 13 and 34% larger than the BPPV and Lesion groups, respectively. However, the sensitivity and specificity at detecting those in the Lesion group was moderate at ~0.5, which was similar to that of htDVA testing. Another difference with the earlier study is that in this study the VOR phase at 1.6 Hz was also useful for detection. At 1.6 Hz, the Non-vestibular group phase led the BPPV and Lesion groups by 12° and 10°, respectively. Presumably at 1 and 1.6 Hz the VOR predominantly contributes to vision stabilization because the other vision stabilizing systems, i.e., OKN and smooth pursuit, break down at higher frequencies and velocities [e.g., (32)]. The VOR gain at 1 Hz was not affected by age and correlated with the minimum vHIT VOR gain, suggesting that the mid-frequency (and high-frequency) VOR gain is relatively stable compared to the low-frequency VOR that is affected by other vision stabilizing systems.

### Transient (Acceleration Steps) Horizontal VOR and OKN

There was a significant difference in the transient time constant between groups. The Lesion group had a time constant ~1.5 s shorter than the BPPV and Non-vestibular groups. The time constant decreased with age and correlated particularly with the minimum vHIT, suggesting that both tests were measuring high-frequency vestibular function. The sensitivity of the time constant measure was high, but it had poor specificity, which was similar to that of physical DHI testing. This is a reasonable conclusion given that both tests use transient/rapid head rotations as the vestibular stimulus. The transient gain was significantly different for rotation toward the excitatory and inhibitory sides. Overall however, this unilateral VOR test failed to detect differences between the ipsilesional and contralesional sides. Transient VOR testing identified one participant with likely central vestibular dysfunction. There was a close to 5% significance difference in OKN time constant between ipsilesional and contralesional whole-body rotations, but it failed to detect a difference between BPPV and Lesion groups.

### Clinical and Video Head Impulse Testing (cHIT and vHIT)

Clinical head impulse testing detected a difference between the Non-vestibular and BPPV/Lesion (combined) groups, but failed to detect a difference between BPPV and Lesion groups. Clinical head impulse testing did not correlate with any of the other tests in this study. The sensitivity of cHIT was low, but it had high specificity. Video head impulse testing detected a significant difference between ipsilesional vs. contralesional gains, but also failed to detect a difference between BPPV and Lesion groups. Similar to cHIT, vHIT had low sensitivity, but high specificity. Video head impulse minimum gain did not change with age, but correlated particularly well with the VOR gain measured during sinusoidal rotations at 1 Hz, suggesting that peripheral vestibular function between mid-frequencies (1 Hz) and high-frequencies [head impulses spectral content ~2.5 Hz, (33)] is generally consistent.

### Summary of Findings per Group and Clinical Relevance

Non-vestibular dizzy patients were best determined by negative cHIT and vHIT gain > 0.75. A low maximum htDVA score (across all six canal plane rotations) was also useful for detecting this group. BPPV patients were best determined by a high maximum htDVA score. Dizzy patients with vestibular cause were best determined by low VOR gain at 1 Hz, large phase lead at 1.6 Hz, or high physical sub-domain DHI score.

For most clinical practices where a rotary chair or vHIT system is unavailable, we suggest the following minimal guidelines to help determine cause of dizziness:

(1) Non-vestibular, if patient has negative cHIT and negative Dix-Hallpike/Roll test nystagmus;
(2) BPPV, if patient has maximum htDVA score > 0.316 (threshold used for analysis above) and positive Dix-Hallpike/Roll test nystagmus;
(3) Vestibular (Lesion), if patient has physical domain DHI score > 5.9 (threshold used for analysis above) and negative Dix-Hallpike/Roll test nystagmus.

To our knowledge, the present study is the largest that has used a panel of experts to examine a patient's history as well as their responses to physiological, cardiovascular, and vestibular tests, to classify their cause of dizziness and then retrospectively examine the performance of each individual vestibular test in correctly classifying the patient. The larger sample size of the present study (*n* = 193), compared to the earlier study (*n* = 76), allowed us to report here sensitivity and specificity measures for each vestibular test as well as determine the effect of age on each test result.

## Conclusion

When the cause of dizziness in participants is assessed by an expert panel evaluating results from a battery of vestibular, cardiovascular, and psychological tests, the results from each vestibular test do not necessarily align with the assigned participant group. Despite the size of this study, the effect size of many of the variables tested were small suggesting generally poor correlations between the test result and group assigned. This study reinforces the idea that one vestibular test alone, e.g., vHIT only, is insufficient to determine the cause of dizziness. Age affected cHIT, htDVA, and transient VOR time constant measures, but did not affect vHIT, DHI, and sinusoidal VOR gain at 1 Hz measures.

The results from this study suggest total physical DHI score and the transient VOR (acceleration step) time constant are best at detecting those in the Lesion group. However, those two tests have low specificity, so they should be used in conjunction with cHIT or vHIT. Sinusoidal VOR gain at 1 Hz and htDVA had similarly moderate sensitivity and specificity. VHIT and htDVA were best at detecting differences between ipsilesional and contralesional sides.

## Data Availability Statement

The raw data supporting the conclusions of this article will be made available by the authors, without undue reservation.

## Ethics Statement

The studies involving human participants were reviewed and approved by Human Research Ethics Committee at the University of New South Wales. The patients/participants provided their written informed consent to participate in this study.

## Author Contributions

WF collected data, processed data, helped with data interpretation, and revised draft manuscript. JM recruited patients, collected data, helped with data interpretation, and revised draft manuscript. AC collected data, processed data, and helped with data interpretation. PH wrote software, collected data, processed data, and helped with data interpretation. SL funded study, helped conceive study, helped with data interpretation, and revised draft manuscript. AM funded study, helped conceive study, main data interpretation, and wrote draft manuscript. All authors contributed to the article and approved the submitted version.

## Conflict of Interest

The authors declare that the research was conducted in the absence of any commercial or financial relationships that could be construed as a potential conflict of interest.
